# Impact of Periprosthetic Fibroblast-Like Cells on Osteoclastogenesis in Co-Culture with Peripheral Blood Mononuclear Cells Varies Depending on Culture System

**DOI:** 10.3390/ijms20102583

**Published:** 2019-05-26

**Authors:** Miriam I. Koehler, Eliza S. Hartmann, Sabine Schluessel, Felicitas Beck, Julia I. Redeker, Baerbel Schmitt, Marina Unger, Martijn van Griensven, Burkhard Summer, Andreas Fottner, Susanne Mayer-Wagner

**Affiliations:** 1 Department of Orthopaedics, Physical Medicine and Rehabilitation, University Hospital, LMU Munich, Marchioninistraße 15, 81377 Munich, Germany; miriam.koehler@uk-essen.de (M.I.K.); eliza.hartmann@insel.ch (E.S.H.); sabine.schluessel@med.uni-muenchen.de (S.S.); huber.feli@googlemail.com (F.B.); Julia.redeker@icloud.com (J.I.R.); baerbel.schmitt@med.uni-muenchen.de (B.S.); andreas.fottner@med.uni-muenchen.de (A.F.); 2 Department of Cardiology and Vascular Medicine, West-German Heart and Vascular Center, University Duisburg-Essen, Hufelandstr. 55, 45147 Essen, Germany; 3 Experimental Trauma Surgery, Department of Trauma Surgery, Klinikum Rechts der Isar, Technical University of Munich, Ismaninger Strasse 22, 81675 Munich, Germany; marina.unger@tum.de (M.U.); martijn.vangriensven@tum.de (M.v.G.); 4 Department of Dermatology, Ludwig-Maximilians-University, Frauenlobstr. 9-11, 80337 Munich, Germany; burkhard.summer@med.uni-muenchen.de

**Keywords:** fibroblasts, osteolysis, osteoclastogenesis, aseptic implant loosening, transwell culture

## Abstract

Co-culture studies investigating the role of periprosthetic fibroblasts (PPFs) in inflammatory osteoclastogenesis reveal contrary results, partly showing an osteoprotective function of fibroblasts and high OPG expression in monolayer. These data disagree with molecular analyses of original periosteolytic tissues. In order to find a more reliable model, PPFs were co-cultivated with peripheral blood mononuclear cells (PBMCs) in a transwell system and compared to conventional monolayer cultures. The gene expression of key regulators of osteoclastogenesis (macrophage colony-stimulating factor (MCSF), receptor activator of NF-κB ligand (RANK-L), osteoprotegerin (OPG), and tumor necrosis factor alpha (TNFα)) as well as the ability of bone resorption were analyzed. In monolayer co-cultures, PPFs executed an osteoprotective function with high OPG-expression, low RANK-L/OPG ratios, and a resulting inhibition of osteolysis even in the presence of MCSF and RANK-L. For transwell co-cultures, profound changes in gene expression, with a more than hundredfold decrease of OPG and a significant upregulation of TNFα were observed. In conclusion, we were able to show that a change of culture conditions towards a transwell system resulted in a considerably more osteoclastogenic gene expression profile, being closer to findings in original periosteolytic tissues. This study therefore presents an interesting approach for a more reliable in vitro model to examine the role of fibroblasts in periprosthetic osteoclastogenesis in the future.

## 1. Introduction

Osteoclastogenesis and thus increased bone resorption is a major complication in diseases involving chronic inflammation. Well known examples are aseptic loosening of orthopedic or dental implants, septic processes like periodontitis as well as autoimmune diseases, e.g., rheumatoid arthritis. Although mediated by different triggers, it is presumed that similar signaling cascades are involved in the differentiation and activation of osteoclasts in these diseases [1,2,3,4,5]. Fibroblasts seem to play a central role in inflammatory bone modeling, as they have been found to release main osteoclastogenic mediators as macrophage colony-stimulating factor (MCSF), receptor activator of NF-κB ligand (RANK-L), and tumor necrosis factor alpha (TNFα), as well as osteoprotegerin (OPG) [6,7,8,9].

This work focuses on the pathophysiological processes involved in aseptic loosening of hip and knee arthroplasties, which is one of the major causes of late implant failure, represents the main reason for revision surgeries [10] and is associated with worse functional outcomes [11,12]. It is known that, by inflammatory responses to wear particles and altered biomechanical conditions, signaling cascades are set in motion within the periprosthetic tissue resulting in the differentiation and activation of osteoclasts [4,13,14]. Analyzing periprosthetic tissues of aseptically loosened implants has revealed elevated gene expression and protein levels of MCSF [15], RANK-L [16,17,18] and TNFα [19] compared to negative controls. OPG, on the other hand, has been described as stable [16,17] or downregulated [15,18,20,21], resulting in high RANK-L/OPG ratios [16,17,18,21].

In vitro, co-cultures of peripheral blood mononuclear cells (PBMCs) and fibroblast-like cells are a commonly used model to examine the role of human fibroblasts in the differentiation and activation of osteoclasts in inflammatory osteolysis [6,8,22,23,24,25,26]. Regarding the fibroblasts’ capacity to promote osteoclastogenesis, the studies reveal a broad spectrum of partly conflicting results. If gene expression data of co-cultures were obtained, high levels of OPG and resulting low RANK-L/OPG ratios are reported [23,25,26], suggesting an osteoprotective function of fibroblasts in these co-cultures. These data conflict with the results of the gene expression analyses of original inflammatory periosteolytic tissues mentioned above.

Regarding the overall inhomogeneous results of these studies, partly contradicting findings in periosteolytic tissues, it has to be questioned whether the established co-culture model is suitable to analyze the role of fibroblasts in osteoclastogenesis.

In order to optimize the existing cell culture model towards a still practicable but more reliable system, we chose a transwell cell culture model, in which cells form conglomerates on the permeable membranes of transwell inserts. Compared to conventional monolayers, transwell cultures have advantages regarding an increased area of intercellular contact, reduced exposure to foreign material and improved access to cell culture medium [27].

The aim of this study was to determine the effect of an alteration of co-culture conditions from a conventional monolayer model towards a transwell system on the osteoclastogenic potential of fibroblasts. For this purpose, we cultivated periprosthetic fibroblast-like cells (PPFs), obtained from patients with aseptic loosening of hip- or knee-arthroplasties, in the presence of PBMCs on conventional plates as well as in transwell inserts. The co-cultures were then compared regarding gene expression of the main mediators of osteoclastogenesis and the formation of resorbing osteoclasts.

## 2. Results

### 2.1. TRAP and Hoechst Staining of Monolayer Cultures

**Figure 1:**
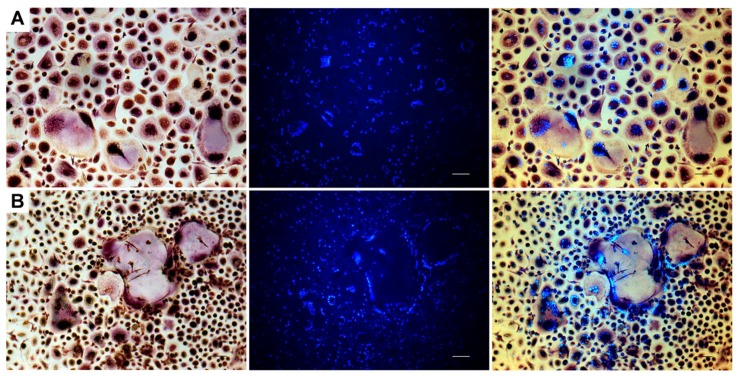
Tartrate resistant acid phosphatase (TRAP) and Hoechst staining of negative and positive controls on day 28. Peripheral blood mononuclear cells (PBMCs) cultivated without (**A**) and with (**B**) additional macrophage colony-stimulating factor (MCSF) and receptor activator of NF-κB ligand (RANK-L) respectively stained for TRAP and with Hoechst. Photos were taken using a light microscope (left), a fluorescence filter (middle), and a combination of both (right). TRAP positive multinucleated cells (MNCs) of various sizes, irrespective of the presence of MCSF- and RANK-L. Bar represents 100 µm.

**Figure 2:**
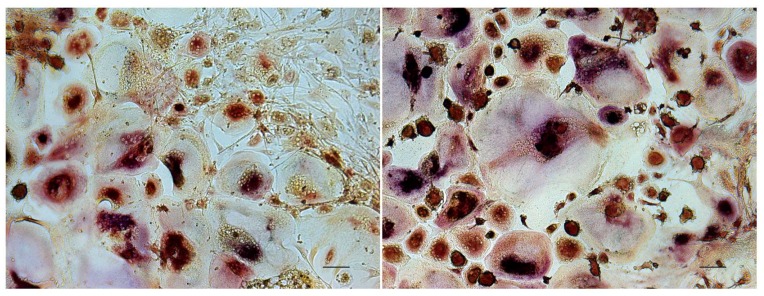
TRAP staining of PBMC/PPF (periprosthetic fibroblast-like cells) co-cultures on conventional culture plates on day 28. TRAP positive MNCs of various sizes surrounded by TRAP negative, spindle-shaped cells. Bar represents 50 µm.

The presence of tartrate resistant acid phosphatase (TRAP) positive, multinucleated cells (MNCs) was assessed in conventional 24-well (monolayer) cultures using Hoechst and a TRAP detection kit. Monocultures of PPFs showed all TRAP negative, mononuclear, spindle-shaped cells, irrespective of whether they were cultivated with normal culture medium or stimulated with MCSF and RANK-L. In all cell cultures containing PBMCs, TRAP positive MNCs of various sizes were detected (Figure 1 and Figure 2). In co-cultures, MNCs were surrounded by spindle-shaped, TRAP negative cells (Figure 2).

### 2.2. Resorption Assay on Dentin

**Figure 3:**
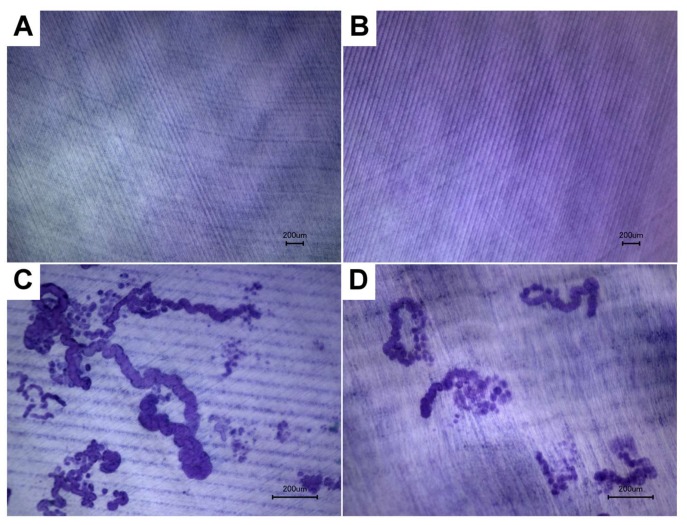
Resorption lacunae on dentin after staining with toluidine blue. Lack of resorption pits in monolayer PBMC/PPF co-cultures (**A**), even when cultivated in the presence of additional MCSF and RANK-L (**B**). PBMC monocultures stimulated with MCSF and RANK-L, cultivated in monolayer (**C**) or on transwell membranes (and transferred on dentin, (**D**)), showed extensive traces of osteolysis.

Resorptive activity was proven by toluidine blue staining of osteolytic lacunae on dentin. On day 29, the positive controls, i.e., PBMC monocultures stimulated with MCSF and RANK-L, showed extensive traces of osteolysis on dentin, regardless of whether they had been cultivated in monolayer (Figure 3C) or on transwell membranes and transferred onto dentin later (Figure 3D). PBMCs in negative controls, that had been cultivated without the addition of MCSF and RANK-L, were not able to resorb bone despite the presence of TRAP positive MNCs. Also, no evidence of osteolysis was found in PPF monocultures and PPF/PBMC co-cultures, irrespective of the culture system (Figure 3A, PPF/PBMC co-culture in monolayer). PBMCs that had been co-cultivated with PPFs in the presence of MCSF and RANK-L on dentin also did not show any sign of lacunar resorption (Figure 3B).

Concerning the density and distribution of nuclei, visualized by Hoechst staining, cultures on dentin showed patterns similar to the correspondent monolayer cultures: in PPF monocultures, nuclei were located isolated from each other; in PBMC-containing cultures, conglomerations of nuclei suggested the presence of MNCs.

### 2.3. Quantitative Real Time PCR

We isolated RNA of PPFs, PBMCs, and co-cultures in monolayer and transwell plates on days 0, 13, and 20. We determined the gene expressions of MCSF, RANK-L, OPG, and TNFα.

#### 2.3.1. Gene Expression in Monolayer Cultures

**Figure 4:**
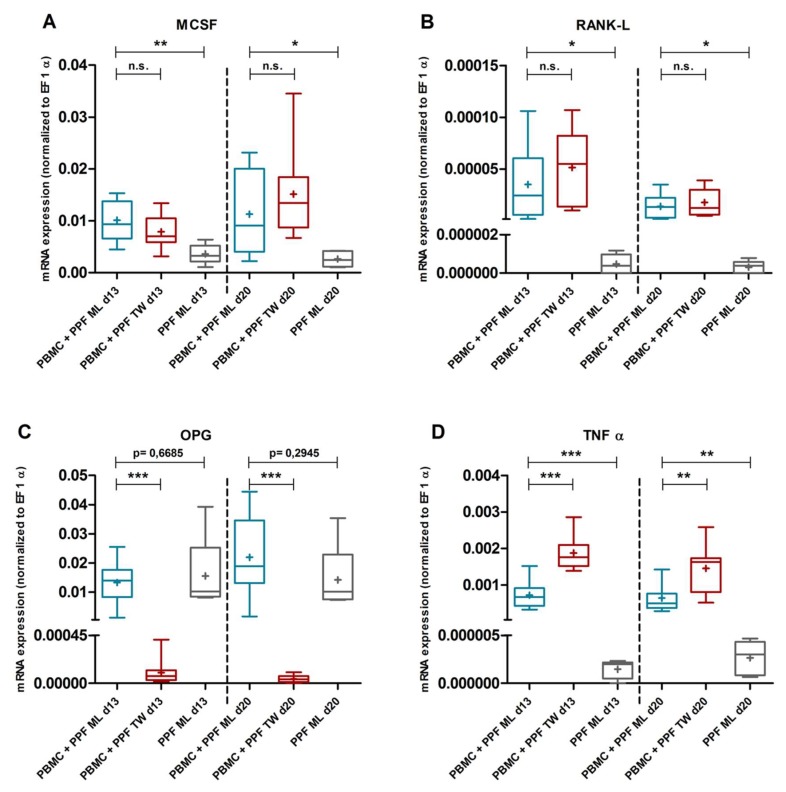
mRNA expression of PPF monocultures and PPF/PBMC co-cultures. Relative mRNA expression (normalized to housekeeping gene EF 1 α) of MCSF (**A**), RANK-L (**B**), OPG (**C**), and TNFα (**D**) in co-cultures of PPFs and PBMCs in monolayer (ML, *n* = 10) and transwell (TW, *n* = 10) as well as in monocultures of PPFs in monolayer (*n* = 5) on d13 and d20. Bands inside the boxes represent group medians, crosses indicate group means. End of whiskers represent minimum and maximum values. *p* values are indicated with * (*p* ≤ 0.05), ** (*p* ≤ 0.01), and *** (*p* ≤ 0.001).

Compared to PPF monocultures, we observed altered expression profiles in PPF/PBMC co-cultures on days 13 and 20 in monolayer. The expression of MCSF, RANK-L and TNFα was significantly higher in co-cultures on both days (Figure 4A,B,D, MCSF d13: *p* = 0.0038, d20: *p* = 0.0319; RANK-L d13: *p* = 0.0497, d20: *p* = 0.0173; TNFα d13: *p* = 0.0006, d20: *p* = 0.0050). OPG expression on the other hand, was already high in PPF monocultures and was not altered by co-culture conditions (Figure 4C, d13: *p* = 0.6685, d20: *p* = 0.2945). The resulting RANK-L/OPG ratio in monolayer co-cultures was low and even decreasing over time. As PBMC monocultures expressed considerably more MCSF, RANK-L, and TNFα than PPF monocultures, they probably contributed significantly to the higher mRNA-levels of these genes in monolayer co-cultures. OPG, however, was hardly expressed in PBMC monocultures (Supplementary Materials Figure S1).

#### 2.3.2. Comparison of Gene Expression in Monolayer and Transwell Co-Cultures

**Figure 5:**
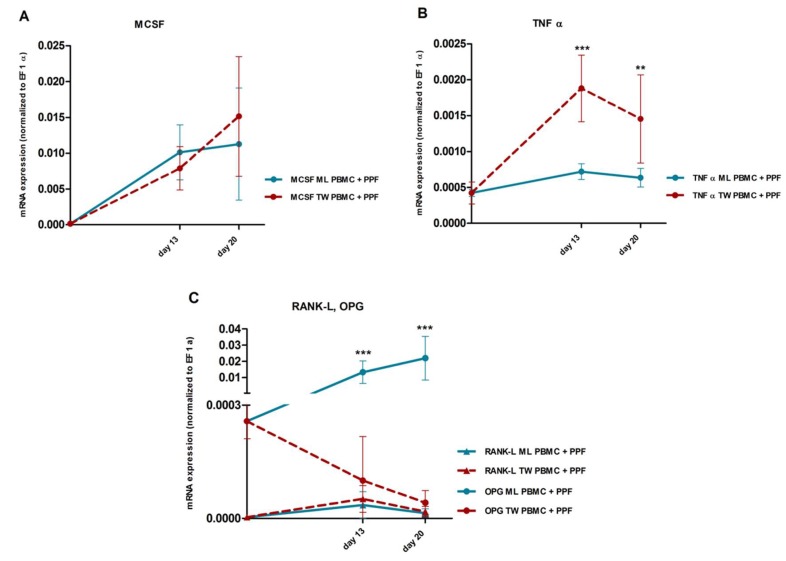
mRNA expression of PPF/PBMC co-cultures in monolayer and transwell over time. Relative mRNA expression (mean and standard deviation, normalized to housekeeping gene EF 1 α) of MCSF (**A**), TNFα (**B**), RANK-L, and OPG (**C**) over time (day 0, 13, 20) of co-cultures of PBMCs and PPFs in monolayer (ML, *n* = 10) and transwell (TW, *n* = 10) culture systems. Significance of differences between monolayer and transwell co-cultures is indicated with * (*p* ≤ 0.05), ** (*p* ≤ 0.01), *** (*p* ≤ 0.001).

Compared to monolayer co-cultures, in transwell co-cultures gene expression was found to be significantly higher for TNFα (Figure 4D and Figure 5B, d13: *p* < 0.0001, d20: *p* = 0.0025) and significantly lower for OPG (Figure 4C and Figure 5C, d13: *p* < 0.0001, d20: *p* < 0.0001). Regarding RANK-L and MCSF, there were no significant differences between the two co-culture systems (Figure 4A,B, and Figure 5A,C, MCSF d13: *p* = 0.1661, d20: *p* = 0.2995; RANK-L d13: *p* = 0.3138, d20: *p* = 0.4864). 

Remarkably, OPG expression in transwell co-cultures was downregulated more than a hundredfold compared to monolayer co-cultures (mean quotient transwell/monolayer d13: 0.008, d20: 0.002) resulting in considerably higher RANK-L/OPG ratios in transwell co-cultures. Also, while the OPG expression in monolayer co-cultures increased over time, the OPG expression in transwell co-cultures even decreased from day 13 to day 20, leading to increasing RANK-L/OPG ratios in transwell culture systems over time (Figure 5C).

## 3. Discussion

In order to find a reliable cell culture model to investigate the role of fibroblasts in osteoclastogenesis in inflammatory processes, we co-cultivated PPFs and PBMCs on conventional monolayer plates and in transwell inserts. In monolayer cultures, fibroblasts exerted an osteoprotective function by producing high levels of OPG. Even in the presence of MCSF and RANK-L, a complete differentiation towards mature, resorbing osteoclasts was inhibited by PPFs in monolayer co-cultures. In transwell co-cultures, however, we observed a significantly higher expression of TNFα and an extensive downregulation of OPG, leading to considerably higher RANK-L/OPG ratios. Despite the significantly more osteoclastogenic gene expression profile in transwell co-cultures, evidence of lacunar resorption could not be found in this study.

MCSF and RANK-L are considered to be the main factors in the direct induction of osteoclastogenesis [5,28,29,30]. First described by Quinn et al. [31], cultivating PBMCs in the presence of MCSF and RANK-L leads to the formation of mature, multinucleated osteoclasts capable of bone resorption. OPG, as a decoy receptor of RANK-L, prevents its binding to its receptor RANK, which is located on osteoclast precursors. It therefore acts as a functional antagonist of RANK-L [5,28,32]. The RANK-L/OPG ratio is considered to be an important indicator for the presence of either osteoclastogenic or osteoprotective conditions [33,34,35]. 

Regarding gene expression in monolayer co-cultures, on the one hand, we were able to show an increased expression of the pro-osteoclastogenic factors MCSF, RANK-L, and TNFα in co-cultures of PPFs and PBMCs. On the other hand, OPG-expression was already high in PPF monocultures and was not altered by co-culture conditions, resulting in low RANK-L/OPG-ratios, even decreasing over time (Figure 4C and Figure 5C). Consistent with our findings, Bloemen et al. describe a significant upregulation of MCSF, RANK-L, and TNFα in co-cultures of periodontal ligament fibroblasts and PBMCs [25]. Also matching our results, persistently elevated OPG levels and low RANK-L/OPG ratios in co-cultures of PBMCs and fibroblasts are reported [23,25]. Because of the high OPG-expression, both in previous studies and in our monolayer co-cultures, we assumed that the absence of complete osteoclastogenesis in our monolayer co-cultures was caused by an actively osteoprotective function of fibroblasts. 

The key role of OPG in the prevention of osteolysis in monolayer co-cultures was confirmed by adding PPFs to cultures of PBMCs on dentin in the presence of MCSF and RANK-L. In contrast to positive controls, where cultivating PBMCs in the presence of MCSF and RANK-L lead to the formation of resorbing osteoclasts, the addition of PPFs resulted in the prevention of lacunar resorption. This suggests that the high levels of OPG expressed by the PPFs were even sufficient to eliminate the effect of the supplemented RANK-L. De Vries et al. [23] describe a similar experiment, also observing high levels of OPG and low RANK-L/OPG ratios in co-cultures of PBMCs and periodontal or gingival fibroblasts. In this study, the inhibitory effect of OPG could be overcome by adding MCSF and RANK-L at higher concentrations (25 ng/mL MCSF and 40 ng/mL RANK-L throughout the whole culture period). This supports the hypothesis that the lack of osteoclast formation in our monolayer co-cultures was not mainly caused by an insufficient expression of MCSF and RANK-L but that osteoclastogenesis was actively inhibited by fibroblasts by producing high levels of OPG.

In accordance with this, a formation of resorbing osteoclasts in monolayer co-cultures of human fibroblasts and PBMCs without the presence of additional factors has rarely been described. Only Costa-Rodrigues et al. observed a complete differentiation of PBMCs towards active osteoclasts by cultivating them in the presence of conditioned media of skin or gingival fibroblasts. It has to be noted, however, that in these experiments, lacunar resorption was found even in negative controls of PBMCs cultivated in base medium in the absence of growth factors or conditioned medium [26]. In other studies, complete osteoclastogenesis has only been achieved in co-cultures stimulated with MCSF and RANK-L [23,25], MCSF [8,24], 1,25(OH)_2_VitD_3_ and Dexamethasone [6], or 1,25(OH)_2_VitD_3_ and MCSF [22]. Quinn et al. [6] and Takayanagi et al. [22] were able to show that, in contrast to unstimulated co-cultures (skin fibroblasts [6] or synovial fibroblasts from patients with rheumatoid arthritis [22] and PBMCs), the addition of 1,25(OH)_2_VitD_3_ and Dexamethasone or 1,25(OH)_2_VitD_3_ and MCSF respectively lead to an increased RANK-L/OPG ratio. Complete osteoclastogenesis was observed only in stimulated co-cultures. Sabokbar et al. [8] describe that only part of the co-cultures of periprosthetic fibroblasts and PBMCs contained resorbing osteoclasts after cultivating them in the presence of MCSF. Interestingly, in those co-cultures that showed successful osteoclastogenesis, the RANK-L/OPG ratio had been higher in the first place, compared to those lacking resorptive activity. Elevated RANK-L/OPG ratios might therefore be a key requirement for osteoclastogenesis in co-cultures of fibroblasts and PBMCs.

In addition to the RANK-L-OPG-axis, the role of TNFα in osteoclastogenesis in PPF/PBMC co-cultures has to be taken into account, too. TNFα has been discussed as a direct mediator of osteoclastogenesis and a possible replacement for RANK-L. Several authors were able to show a RANK-L-independent formation of osteoclasts in the presence of MCSF and TNFα [36,37,38,39,40]. Considering this alternative pathway of a direct induction of osteoclastogenesis via MCSF and TNFα, it has to be assumed that in this study expression of TNFα must have been insufficient to induce a RANK-L-independent osteoclast formation in the monolayer co-cultures. 

We therefore presume that in monolayer co-cultures, the expression of TNFα was not sufficient to induce a RANK-L-independent osteoclastogenesis and that high levels of OPG actively prevented the formation of active osteoclasts in spite of an addition of MCSF and RANK-L. 

Comparing the expression profiles of monolayer co-cultures to those of molecular analyses of periosteolytic tissues, the high levels of OPG described in co-culture models conflict with the findings in original tissues, where mostly low OPG levels and correspondently high RANK-L/OPG ratios are found [16,17,18,21]. A possible reason for these contrary results of original tissues and co-culture-analyses could be that a monolayer co-culture does not sufficiently imitate in vivo conditions to be an adequate research model for fibroblast-associated osteoclastogenesis. 

In vivo, cells are surrounded by other cells and extracellular matrix in tissues of three-dimensional structure. In the established co-culture models, however, cells grow in monolayers and cell contact is limited to the lateral cell parts. In several in vitro studies, it has been shown that fibroblasts change the expression of genes involved in osteoclastogenesis when subjected to altered environments, e.g., different culture media or biomechanical forces [6,22,41,42,43]. 

Considering this instability in expression patterns of fibroblasts it may be presumed that every culture model bears a risk to cause changes in cell characteristics. Therefore, to further investigate the molecular processes underlying fibroblast-induced osteolysis and to identify possible targets of therapy, it would be desirable to have a reliable in vitro model, in which cell characteristics are as close to those in vivo as possible while still being feasible with reasonable effort.

In contrast to monolayer cultures, in so-called transwell systems cells grow on permeable membranes. Sabater et al. were able to show that cultivating fibroblasts in transwell systems leads to an increased number of cells and a higher cell mass compared to cultures on standard well bottoms, despite the smaller surface of the transwell membranes [27]. Reaching higher cell densities [27], fibroblasts on transwell membranes grow in conglomerates with a partially three-dimensional, multilayered structure, as shown by our work group in previous experiments with PPF in transwell cultures (Supplementary Materials Figure S2, [44]). This should lead to an increased area of intercellular contact which enforces intercellular connections and reduces exposure to foreign surfaces. The permeable membrane provides the cells with an improved access to cell culture medium. In addition, the altered biomechanical conditions of a transwell culture, e.g., concerning matrix-elasticity or surface stiffness, can certainly contribute to a change of cell characteristics [45].

In this study, significant changes in gene expression of two major mediators of osteoclastogenesis were detected in transwell co-cultures compared to those in monolayer. While we did not observe significant changes in MCSF and RANK-L expression, TNFα was significantly upregulated in transwell co-cultures. Even more remarkably, transwell co-cultures showed an extensive downregulation of OPG, with OPG-levels at least a hundredfold lower than in monolayer co-cultures and even further declining over time. Based on the expression data of PPF and PBMC monocultures (Figure 4C,D, Supplementary Materials Figure S1) it might be assumed, although speculative, that the higher OPG expression is mainly caused by PPFs, whereas PBMCs are supposed to be responsible for most of the TNFα expression. Only by altering the cell culture system and without any additional stimulation, we induced significant changes in the expression of TNFα and OPG, both factors we assumed to be responsible for the prevention of osteoclastogenesis in the monolayer co-cultures. We also observed an impressive increase in the resulting RANK-L/OPG ratio, leading to a more osteoclastogenic environment and better reflecting the conditions found in original inflammatory periosteolytic tissues.

To our knowledge, we are the first work group describing a transwell co-culture system as a possible approach to find a reliable in vitro model to further investigate pathophysiological processes of fibroblast-induced osteoclastogenesis. As this is a first experiment to evaluate the impact of a transwell system on co-cultures of PPFs and PBMCs the study has some limitations.

Despite the significant changes in gene expression in the transwell model, lacunar resorption was observed in neither of the co-cultures. There are several possible reasons for the fact that, in spite of a significant upregulation of TNFα and downregulation of OPG respectively in transwell co-cultures, evidence of a formation of resorbing osteoclasts could still not be found. On the one hand, it cannot be ruled out that expression levels of TNFα or the RANK-L/OPG ratio were still too low to provide a basis for complete osteoclastogenesis. On the other hand, the expression of MCSF, a protein that is an essential factor in both pathways of direct induction of osteoclastogenesis, either via RANK-L or TNFα, was not altered by changing the culture conditions. The possibility of an insufficient intrinsic production of MCSF by fibroblasts in transwell co-cultures does, however, not contradict the hypothesis that fibroblasts can induce complete osteoclastogenesis in osteoclast precursors in inflammatory tissues in vivo. Itonaga et al. showed that arthroplasty derived macrophages differentiate into osteoclasts only by addition of RANK-L as their intrinsic production of MCSF is considerably higher and apparently sufficient for osteoclastogenesis [46]. Therefore, it is possible that, in inflammatory processes in vivo, local monocytes release the main part of MCSF needed for the formation of resorbing osteoclasts. To further investigate this, transwell co-cultures either using monocytes isolated from periosteolytic inflammatory tissues instead of buffy coats or the external supplementation of MCSF could lead to an improvement of the cell-culture model. In addition to this, it cannot be taken for granted that changes in mRNA-expression led to a corresponding alteration of protein levels. However, in monolayer co-cultures stimulated with MCSF and RANK-L the prevention of osteoclastogenesis makes it seem likely that protein levels of OPG correlated well with the upregulated mRNA-expression. Nevertheless, including protein analysis in future studies might provide valuable information in order to understand the underlying molecular processes.

Another limitation might be the limited number of patients whose tissues were used to isolate PPFs from in this first study. Nevertheless, our results were consistent and, considering the main findings of changes in gene expression of TNFα and OPG, significant. Moreover, to exclude any bias by donor-specific characteristics of PBMCs, two PBMC donors were used and PPFs of each patient were co-cultivated with PBMCs of each donor separately. Although of high importance, types of implant bearing surfaces were not the focus of this analysis. We did not differentiate between the types of prostheses (hip or knee prostheses of different material, cemented or uncemented) the PPFs originated from, as it was the main goal to find a better co-culture model to investigate the PPFs’ impact on osteoclastogenesis in general.

One finding that might also raise questions is the fact that we found TRAP positive MNCs in all cell cultures containing PBMCs and that the detection of these cells was no predictor of the actual presence of resorbing osteoclasts. This phenomenon has, however, been repeatedly described in previous studies [23,31,40]. De Vries et al., for example, presented similar findings in co-cultures of periodontal ligament fibroblasts and PBMCs [23]. It is known that neither TRAP nor multinuclearity are specific markers for osteoclasts and have been found in many other cell types [47,48,49,50], leaving lacunar resorption, as a specific functional characteristic, as the most reliable evidence of complete osteoclastogenesis. Therefore, we assume that the non-resorbing, TRAP positive MNCs we observed were either macrophage polycarions or, as described by de Vries et al., osteoclast-like cells, which would have needed further stimulation to develop the ability to actually resorb bone [23].

In conclusion, we showed that PPFs in a monolayer co-culture model actively inhibited the formation of resorbing osteoclasts, presumably by producing high levels of OPG, even eliminating the effect of additionally supplemented RANK-L. We found low RANK-L/OPG ratios in monolayer co-cultures that conflict with the results of molecular analyses of periosteolytic inflammatory tissues. These findings question the appropriateness of the currently used monolayer co-cultures to further investigate the mechanisms of fibroblast-induced osteoclastogenesis. In this study, PPFs and PBMCs were co-cultured in transwell inserts in order to establish a more reliable model. This change of culture conditions induced a profound alteration in the gene expression profile towards a considerably more osteoclastogenic environment in transwell co-cultures, which better reflects the findings in original periosteolytic tissues. Remarkably, OPG expression was reduced more than a hundredfold, resulting in significantly lower RANK-L/OPG ratios. 

With co-culturing PPFs and PBMCs in transwell inserts, we therefore present an interesting approach to study fibroblast-induced inflammatory osteoclastogenesis in a cell-culture model mimicking in vivo findings more closely and hereby laid the groundwork for further research.

## 4. Materials and Methods

### 4.1. Patients

Periprosthetic tissue was obtained from 5 patients (2 male, 3 female; mean age 71 years; range 56–79 years) undergoing revision surgery due to aseptic loosening of hip (*n* = 3) or knee (*n* = 2) endoprotheses in the hospital of the Ludwig Maximilian University, Munich (LMU) in 2011 and 2012. The patients had no history of rheumatologic conditions, allergies to components of the endoprosthetic material or disorders of bone metabolism. Septic loosening was excluded by performing microbiological cultures in the department of Microbiology of the LMU. The study was approved by the LMU medical ethics committee. Based on the study design using disposable material no patient consent was required.

### 4.2. Cell Culture

If not stated otherwise, we used α-Minimal Essential Medium (α-MEM) supplemented with 10% fetal calf serum, 2 mM l-glutamine, 60 IU/mL penicillin, 60 µg/mL streptomycin (all Biochrom, Berlin, Germany) and 0.075 µg/mL amphotericin B (Sigma-Aldrich Co., St. Louis, MO, USA) as culture medium. Cells were cultivated at 37 °C and 5% CO_2_.

### 4.3. Isolation of PPFs

In the operating room, periprosthetic tissue was collected in Dulbecco´s Minimal Essential Medium (DMEM, Biochrom, Berlin, Germany) with 60 IU/mL penicillin, 60 µg/mL streptomycin and 0.075 µg/mL amphotericin B under sterile conditions. PPFs were isolated following a protocol published by Sabokbar et al. [8]: The tissue was rinsed with phosphate buffered saline (PBS, Biochrom, Berlin, Germany), cut in pieces of 1–3 mm and incubated for 15 min in DMEM supplemented with 60 IU/mL penicillin, 60 µg/mL streptomycin and 0.25 µg/mL amphotericin B at room temperature (RT). After rinsing with PBS, the tissue was digested in 0.1% Collagenase (Sigma-Aldrich Co., St. Louis, MO, USA) in DMEM for 1 h at 37 °C. The fluid phase was then pipetted into a 50 mL falcon via a cell strainer (pore size 70 µm, both BD Bioscience, San Jose, CA, USA). The remaining tissue was incubated with Versene (Invitrogen, Darmstadt, Germany) at 37 °C for another hour before being added to the 50 mL falcon via the cell strainer. After centrifugation at 1500 rpm (Multifuge 1L-R, Eppendorf, Hamburg, Germany) for 5 min, the cell pellet was repeatedly washed with PBS, resuspended in culture medium and cultivated in T75 culture flasks (Nunc, Roskilde, Denmark). Medium was changed twice a week. Cells were passaged at 90% confluence. For the experiments, we used PPFs in passage 3 or 4. In a previous study, the PPFs we used in this setting stained positive for S100A4 and were further characterized by molecular expression markers as fibroblast-like cells [44]. 

### 4.4. Isolation of PBMCs

Buffy coats (*n* = 2, male donors, blood group A and 0 respectively, rhesus positive) were obtained from the German Red Cross Blood Donor Service at the university of Ulm, Germany. Buffy coats were diluted with PBS, layered on a density gradient medium (Biocoll, Biochrom, Berlin, Germany) and centrifuged for 30 min at 2000 rpm with the brake off. The interphase was collected, diluted in PBS and centrifuged at 1200 rpm for 10 min. The cell pellet was then repeatedly washed with PBS and resuspended in culture medium. Cells were used for experiments in passage 0.

### 4.5. Culture Conditions 

Cells were cultivated on conventional 24-well plates for adherent cells (Nunc, Roskilde, Denmark) and on the membranes of 24-transwell plate inserts (pore size 0.4 µm, Nunc, Roskilde, Denmark). Cell densities in both culture systems were 1.2 × 10^5^ PPFs per well and 6 × 10^6^ PBMCs per well respectively. Cells were cultivated for 13, 20, 28, and 29 days. The culture medium was changed three times a week.

Experiments were performed in monocultures of PPFs as well as in co-cultures of PPFs and PBMCs. In co-cultures, PBMCs were seeded on day 0 and medium was changed on day 1 to remove non-adherent cells. PPFs were added on day 3. To avoid any bias by donor specific cell characteristics in co-cultures, PPFs of each patient were co-cultivated with PBMCs of two different donors separately. All cell culture groups were cultivated both in conventional 24-well-plates and in transwell inserts. In monolayer, we additionally performed monocultures of PPFs and co-cultures of PPFs and PBMCs in the presence of MCSF and RANK-L. 

### 4.6. Control Groups

As positive and negative controls of osteoclast formation, we used monocultures of PBMCs with and without the addition of MCSF and RANK-L respectively on conventional plates and in transwell inserts.

### 4.7. Stimulation with MCSF and RANK-L

In stimulated cultures, MCSF (recombinant human MCSF, R&D Systems, Minneapolis, MN, USA) and RANK-L (recombinant human sRANK Ligand, Peprotech, Rocky Hill, NJ, USA) were added to the culture medium as follows: on days 0, 1, and 3, 25 ng/mL MCSF were added. On day 6, half of the medium was replaced by culture medium containing 20 ng/mL RANK-L. From then on, 20 ng/mL RANK-L were added to the culture medium every time it was changed.

### 4.8. TRAP and Hoechst Staining of Monolayer Cultures

On day 28, cell cultures in conventional 24-well plates were stained with Hoechst (Hoechst 33342, Invitrogen, Darmstadt, Germany) and for TRAP, using a commercial kit (Sigma-Aldrich Co., St. Louis, MO, USA) to detect TRAP positive MNCs. The cells were rinsed with PBS and fixated on the bottoms of the wells with acetone and a citrate solution according to the manufacturer´s instruction. After repeatedly rinsing with aqua dest and air-drying the plates, the bottoms were covered with staining solution and incubated for 25 min at 37 °C in the dark. The cells were rinsed with aqua dest again and incubated with Hoechst (1:1000 in PBS) for 10 min in the dark at RT. Plates were then scanned for the presence of multinucleated, TRAP positive cells.

### 4.9. Resorption Assay on Dentin

In order to determine the cells´ ability to resorb bone they were cultivated on ivory dentin slices. Pieces of ivory were obtained from German Customs and flat disks were cut (9 mm × 9 mm × 1 mm) using a band saw with a diamond coated cutting band (Exakt Advanced Technologies GmbH, Norderstedt, Germany). After disinfection in 70% Ethanol for 2 days, the slices were placed into the wells of 24-well plates for suspension cells (Sarstedt, Nuembrecht, Germany). Monolayer cultures were seeded on dentin in the above-mentioned densities and cultivated for 29 days. Transwell cultures were trypsinized (0.05% Trypsin and 0.02% ethylenediaminetetraacetic acid (both Biochrom, Berlin, Germany)) on day 21 using a cell scraper (Nunc, Roskilde, Denmark) to remove remaining cells and centrifuged at 1500 rpm for 5 min. Then, cells were resuspended in culture medium, transferred onto dentin and cultivated for another 8 days.

On day 29, the ivory slices were stained with Hoechst for 10 min as described above to evaluate the distribution of nuclei. Traces of lacunar resorption were visualized by staining with toluidine blue: cells were removed by washing the slices with sodium hypochlorite (Merck, Darmstadt, Germany), rinsing them with tap water and then covering them with 80% ethanol for a few seconds. The slices were wiped with a paper towel and stained with 1% toluidine blue (Waldeck, Münster, Germany) for 6–8 s until the dentin had taken on a light blue color. They were rinsed again with tap water and checked for resorption lacunae (BioRevo Fluorescence Microscope, Keyence, Neu-Isenburg, Germany). 

### 4.10. RNA Isolation

RNA was isolated from PPFs, PBMCs, and co-cultures on days 0, 13, and 20 from conventional monolayer and transwell cultures. On day 0, cells were washed with PBS, covered with Qiazol lysis reagent (Qiagen, Venlo, The Netherlands) and stored at –80 °C. On days 13 and 20, cells in monolayer cultures were washed with PBS, suspended in Qiazol lysis reagent and stored at –80 °C. The membranes of transwell cultures were rinsed with PBS, detached from the inserts together with the attached cell pellet, placed in cryo tubes (Thermo Fisher Scientific, Waltham, MA, USA) and frozen in liquid nitrogen. Using a Mikro-Dismembrator S (Sartorius, Göttingen, Germany) the cells and membranes were shredded at 3000 rpm for 1 min and stored in Qiazol lysis reagent at –80 °C. 

Cell lysates were defrosted on ice and 200 µL/mL of chloroform (Sigma-Aldrich Co., St. Louis, MO, USA) were added. Samples were agitated, incubated for 10 min at RT and centrifuged at 15,000 rcf for 30 min at 4 °C. The interphases were collected, and 0.5 mL of isopropanol were added. Samples were mixed on a vortex, incubated at RT for 10 min and centrifuged overnight at rcf at 4 °C. On the next day, supernatants were discarded and, after centrifugation (rcf, 20 min, 4 °C), pellets were washed with 75% ethanol twice and air-dried. After resuspension in PCR grade water (Roche Diagnostics, Mannheim, Germany) RNA-concentrations were measured using a Nano-Drop (Thermo Fisher Scientific, Waltham, MA, USA).

### 4.11. Quantitative Real-Time PCR

0.5 µg of RNA were transcribed into cDNA using a QuantiTect Reverse Transcription Kit (Qiagen, Venlo, The Netherlands). The cDNA was diluted 1:3 in PCR grade water. For quantitative real-time PCR, 2.5 µL of cDNA, 5 µL of SybrGreen I (Roche Diagnostics, Mannheim, Germany) and 2.5 µL of primer solution (300 nM in PCR grade water, Table 1) were pipetted into the wells of LightCycler 480 Multiwell plates 96 (Roche Diagnostics, Mannheim, Germany). In a LightCycler (LightCycler 96 Real-Time PCR System, Roche Diagnostics, Mannheim, Germany), 40 amplification cycles were performed after preincubating the samples at 95 °C for 10 min. Primer sequences, primer concentrations and conditions of amplification can be found in Table 1. Experiments were performed in triplicate. In order to obtain the comparability of mRNA expression of different genes within the graphs, transcription levels of target genes were normalized to the housekeeping gene (HKG) elongation factor 1 α (EF 1 α, [51]) using the following formula (CP: crossing point):

(1) $$\frac{2{\;}^{CP\;\left(HKG\right)}}{2^{\;CP\;\left(target\;gene\right)}}$$

### 4.12. Statistical Analysis

Analyses were performed with GraphPad Prism 5.01 (GraphPad Software, La Jolla, CA, USA, RRID:SCR_002798). For significance testing we used t-tests for unpaired samples. *p* values < 0.05 were considered significant. 

## Figures and Tables

**Table 1:** Primers for quantitative real-time PCR.

Gene	Primer Sequences (5′–3′)	Annealing Temperature (AT)	Amplification (95 °C–AT–72 °C)	Amplicon Size (bp)
EF 1 α ^1^	AGCGCCGGCTATGCCCCTG CTGAACCATCCAGGCCAAAT	60 °C	15 s–60 s–10 s	59
MCSF ^2^	CCGAGGAGGTGTCGGAGTAC AATTTGGCACGAGGTCTCCAT	60 °C	10 s–10 s–15 s	100
RANK-L ^2^	CATCCCATCTGGTTCCCATAA GCCCAACCCCGATCATG	60 °C	10 s–10 s–15 s	60
OPG ^2^	CTGCGCGCTCGTGTTTC ACAGCTGATGAGAGGTTTCTTCGT	60 °C	30 s–60 s–15 s	100
TNFα ^2^	CCCAGGGACCTCTCTCTAATC GCTTGAGGGTTTGCTACAACATG	60 °C	30 s–60 s–15 s	103

^1^ [51], ^2^ [25].

## Supplementary Materials

Supplementary materials can be found at https://www.mdpi.com/1422-0067/20/10/2583/s1.

## Abbreviations

MCSF	Macrophage colony-stimulating factor
RANK-L	Receptor activator of NF-κB ligand
TNFα	Tumor necrosis factor alpha
OPG	Osteoprotegerin
PBMCs	Peripheral blood mononuclear cells
PPFs	Periprosthetic fibroblast-like cells
α-MEM	α-Minimal Essential Medium
DMEM	Dulbecco´s Minimal Essential Medium
PBS	Phosphate buffered saline
RT	Room temperature
TRAP	Tartrate resistant acid phosphatase
MNCs	Multinucleated cells
HKG	Housekeeping gene
EF 1 α	Elongation factor 1 α
CP	Crossing Point
ML	Monolayer
TW	Transwell
