# Supplementary material for: Impact of Periprosthetic Fibroblast-Like Cells on Osteoclastogenesis in Co-Culture with Peripheral Blood Mononuclear Cells Varies Depending on Culture System
Source: Int J Mol Sci. 2019 May 26;20(10):2583. doi: 10.3390/ijms20102583 (PMC6567687; doi:10.3390/ijms20102583)
Supplement: Supplementary file 1 [file ijms-20-02583-s001.pdf]

Supplementary Materials

Figure S1

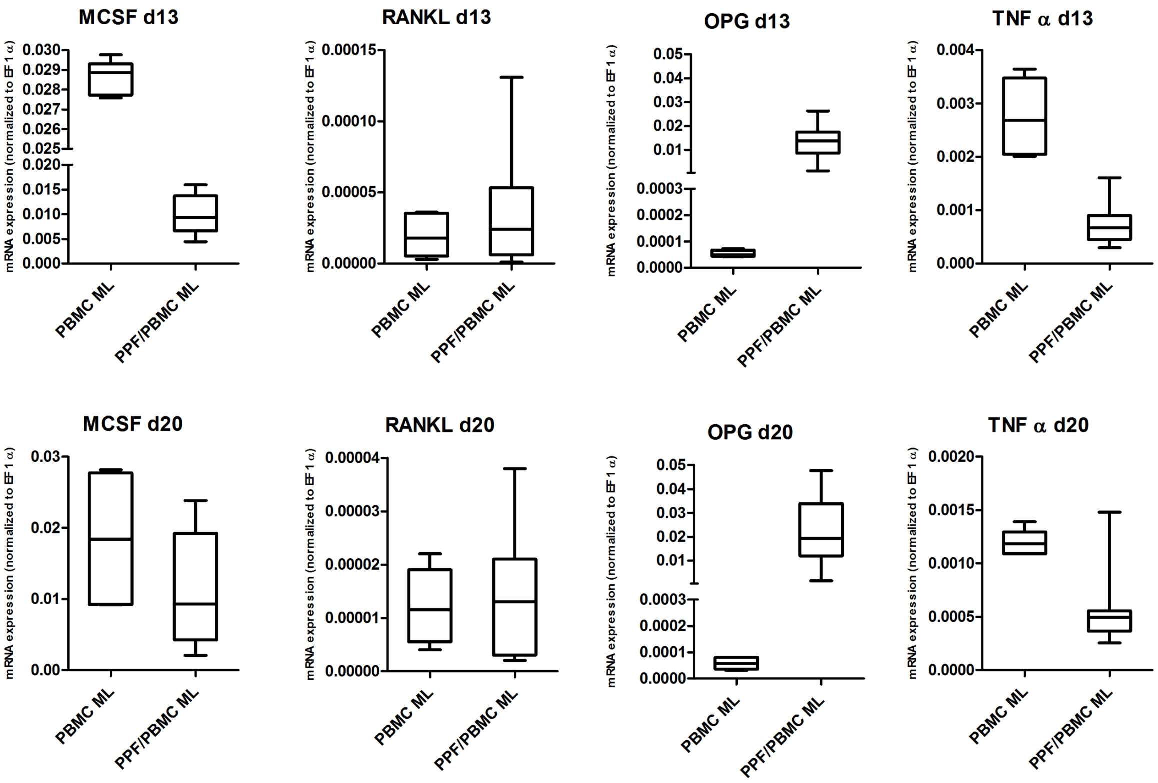

Figure S1. Gene expression of MCSF, RANK-L, OPG and TNFα in monolayer cultures of PBMCs alone and co-cultures of PBMCs and PPFs on days 13 and 20. Apart from OPG, a significant contribution to gene expression levels of co-cultures by PBMCs can be assumed.

Figure S2

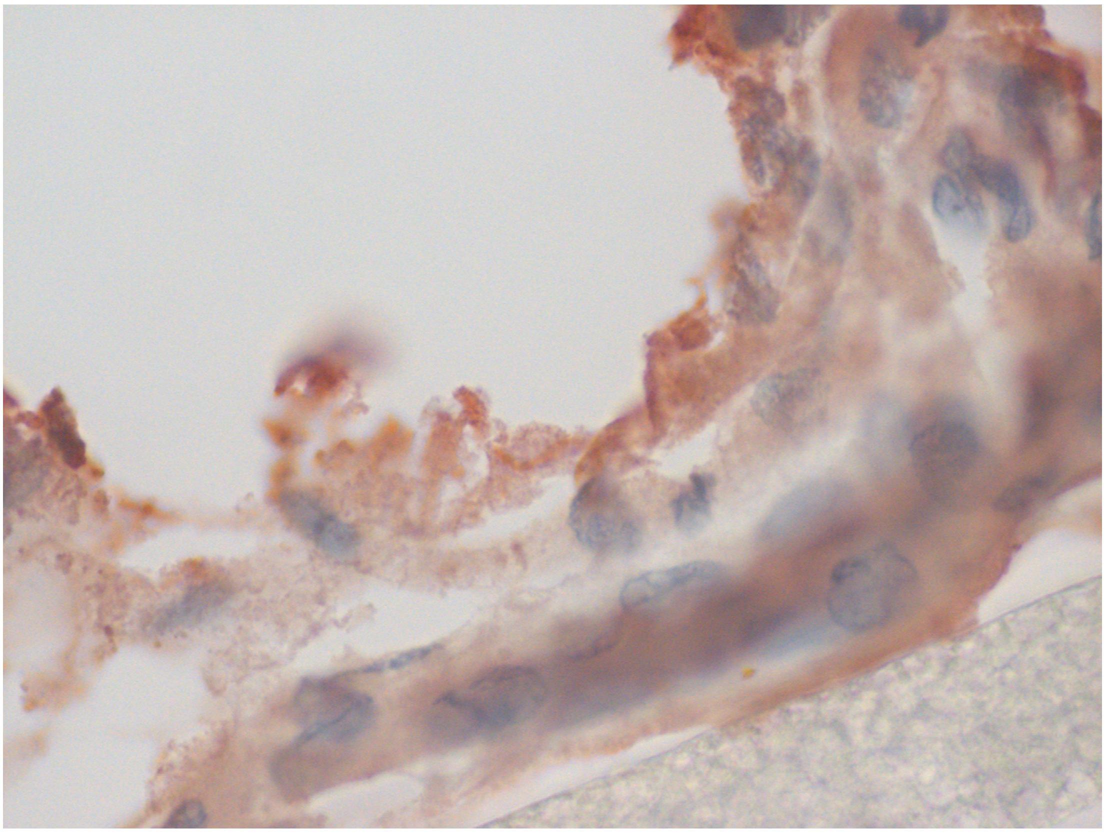

Figure S2. Periprosthetic fibroblasts, staining positive for S100, grow in conglomerates with a partially three-dimensional, multilayered structure on transwell membranes (100x).
